# Advancing research in NeuroAIDS using collaboration and public data sharing

**DOI:** 10.1186/s12920-015-0150-9

**Published:** 2015-11-11

**Authors:** Lucette A. Cysique

**Affiliations:** University of New South Wales, Sydney, Australia; Neuroscience Research, Sydney, Australia; St. Vincent’s Hospital Centre for Applied Medical Research, Sydney, Australia

## Abstract

In this issue of BMC Medical Genomics Griffin et al. present a user-friendly and freely accessible HIV-associated neurocognitive disorder (HAND) genomic database that compiles viral (HIV-1) genetic sequences and other relevant clinical and treatment data. We discuss the benefits and caveats of public data sharing in NeuroAIDS research, while emphasizing the importance of such novel initiatives for advancing knowledge.

## Commentary

Novel research initiatives are needed to alleviate and hopefully eventually cure complex diseases such as HIV-associated neurocognitive disorder (HAND). The paper by Griffin et al. [1] in this issue of BMC Medical Genomics is an example of one such initiative. The authors have developed a HAND genomic database that compiles viral (HIV-1) genetic sequences with information on HAND and other relevant clinical and treatment data (previously collected by a number of investigators who agreed to have the viral sequences compiled). Together with American and Chinese collaborators, the authors of the database developed a user-friendly system that can be utilized by other researchers and further developed. The development stage has not been clearly outlined in this first publication, but the database authors are contactable via the database webpage (http://www.handdatabase.org). And it is expected that as the database evolves new requirements will be developed similarly to already existing HIV database which however do not collect information on HAND [2]. The authors have clearly described their quality control protocol for accuracy of the genetic data. It is assumed that any novel sequences will undergo the same process. They have also used a database interface that is widely accessible (MySQL (v.5.6.17)), similar to other HIV genetic databases [3].

The benefits of such an initiative are immediate: because this is a public data sharing system, of which I along with many other scientists am a strong advocate [4], almost instant inception of new projects is possible. And as noted by the authors the system will foster studies on relatively neglected parts of the HIV-1 genome that are relevant to HIV-1 neuropathogenesis. A further benefit is the increased statistical power that has become a necessary condition for robust genetic analyses allowing the required cross-validation sampling strategy. A freely accessible database will facilitate a better representation of the global HIV epidemic with further potential data addition from researchers in resource-limited settings who are otherwise often indirectly excluded due to financial restrictions.

One important caveat not mentioned in the paper (probably because it was beyond its scope) is the issue of international variability in policies around data sharing [5]. Because HAND and related information are included, I would expect from my own research experience that each investigator wanting to add data to this database will have to obtain ethics approval from their respective institutions. Public data sharing is increasingly common and many universities now seek to facilitate it. But this does not mean that complex standardization issues are resolved [5] especially in the case of a disorder that alters cognitive functioning as its primary endpoint and the need to harmonize data collection for each disease phenotype. Perhaps for the time being the current database developers should specify what security measures have been put in place for the database and whether or not they have a long-term funding horizon, as has been advocated for “omics sharing” [4].

Finally, the authors might include a more refined phenotyping of HAND and in particular one that matches the current diagnostic criteria in any future development of the database [6]. Other information relating to HIV or comorbid brain insult would also be needed, as well as environmental information that is known to impact HAND prevalence and incidence (such as education level) [7]. This may be the occasion for extending collaborations, which may lead for example to new genetic epidemiology research that tackles the complex issue of gene-environment interactions in the development of HAND.

## Disclosures

Dr. Cysique is supported by the National Heath and Medical Research Council of Australia Career Development Fellowship APP1045400 (Cysique CIA/PI). Dr. Cysique received honoraria from Abbvie Ltd, CogState Ltd, ViiV healthcare, partial salary support in 2012 from Mercks Sharp Dome and CogState Ltd. Dr. Cysique has research support from Abbvie Ltd, ViiV healthcare and the Australian National Association of People Living with HIV/AIDS (NAPWA) and Gilead Sciences.
